# About to Burst

**DOI:** 10.4269/ajtmh.14-0473

**Published:** 2015-03-04

**Authors:** Sumontra Chakrabarti, Philippe Garzon, Adam Mohammed, Mahin Baqi, Jay Keystone

**Affiliations:** Infectious Diseases and Tropical Medicine, University of Toronto, Trillium Health Partners - Mississauga Hospital, Mississauga, Ontario, Canada; Hepatobiliary and General Surgery, University of Toronto, Trillium Health Partners - Mississauga Hospital, Mississauga, Ontario, Canada; Thoracic and General Surgery, Trillium Health Partners - Mississauga Hospital, Mississauga, Ontario, Canada; Infectious Diseases, William Osler Health System - Etobicoke General Hospital, Etobicoke, Ontario, Canada; Tropical Medicine, University of Toronto, University Health Network - Toronto General Hospital, Toronto, Ontario, Canada

**Figure 1.**
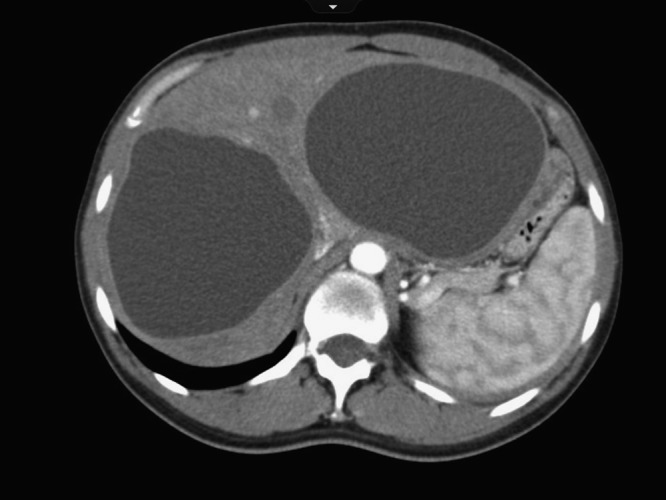
Computed tomography (CT) image of two large, unilocular hepatic hydatid cysts.

This 29-year-old woman from Afghanistan presented with intractable cough. Imaging showed she had a cystic lesion in her lung and two large unilocular cysts consistent with cystic echinococcosis (Figure 1). A day before the surgery, she coughed up copious amounts of white sputum, called vomica, indicating rupture of the lung cyst. It was resected and she was treated with perioperative albendazole and praziquantel.

**Figure 2.**
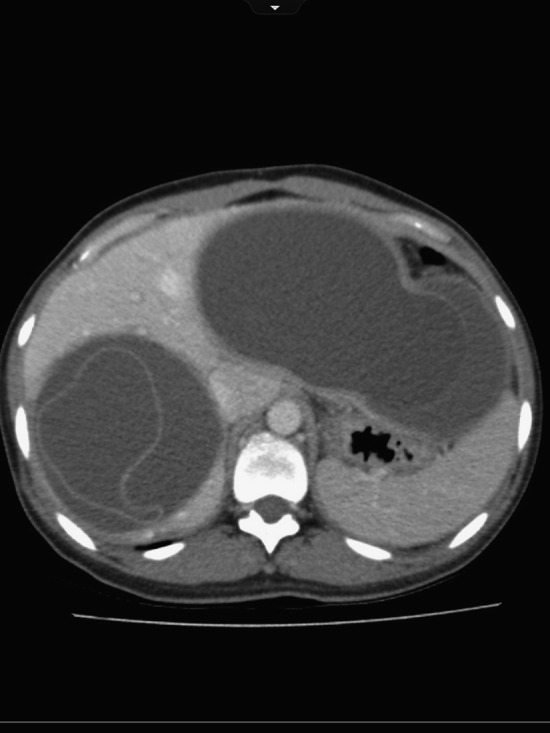
Computed tomography (CT) image of the same two ruptured unilocular hydatid cysts with the clearly visible cyst walls collapsed away from the liver. This is known as the “serpent sign.”

**Figure 3.**
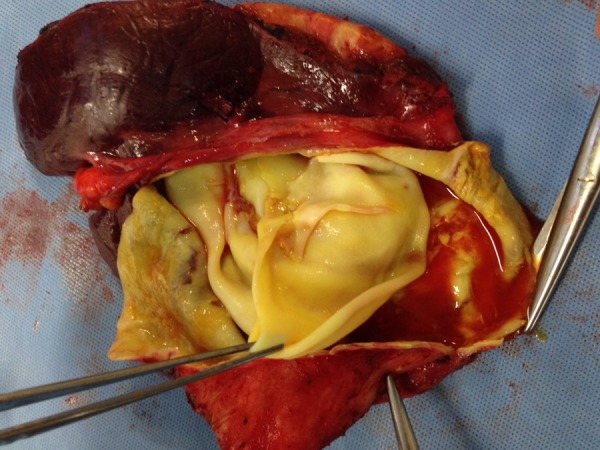
Gross pathology of resected liver with visible jelly-like membrane consistent with hydatid cyst.

We then began to discuss management of the liver cysts, trying to decide between puncture, aspiration, injection, reaspiration (PAIR) or surgical resection.^1^,^3^–^5^ However, during her recovery about 6 weeks after resection of the lung cyst, she presented with abdominal pain. Her abdominal imaging was redone, and the classic signs of ruptured hydatid are seen (Figure 2). Though there is not much evidence on the subject, anecdotally, surgeons describe softening and decrease in turgidity of large hydatid cysts with medical therapy.^2^ Given the almost simultaneous change in both cysts, we postulate that the perioperative antiparasitic therapy for the pulmonary cyst may have been a contributor to the rupture of the large hepatic cysts. The cysts were removed operatively on an emergent basis with no evidence of intraperitoneal spillage (Figure 3). The patient did quite well post-operatively with no recurrence of hydatid disease thus far.
